# Experimental and Theoretical Investigations of Three-Ring Ester/Azomethine Materials

**DOI:** 10.3390/ma15062312

**Published:** 2022-03-21

**Authors:** Fowzia S. Alamro, Nada S. Al-Kadhi, Sobhi M. Gomha, Saheed A. Popoola, Muna S. Khushaim, Omaima A. Alhaddad, Hoda A. Ahmed

**Affiliations:** 1 Department of Chemistry, College of Science, Princess Nourah bint Abdulrahman University, P.O. Box 84428, Riyadh 11671, Saudi Arabia; fsalamro@pnu.edu.sa (F.S.A.); nsalkadhi@pnu.edu.sa (N.S.A.-K.); 2 Chemistry Department, Faculty of Science, Islamic University of Madinah, Madinah 42351, Saudi Arabia; abiodun@iu.edu.sa; 3 Department of Chemistry, Faculty of Science, Cairo University, Cairo 12613, Egypt; 4 Department of Physics, Faculty of Science, Taibah University, P.O. Box 30002, Al-Madina 41447, Saudi Arabia; mkhushaim@taibahu.edu.sa; 5 Nanotechonolgy Center, Taibah University, P.O. Box 30002, Al-Madina 41447, Saudi Arabia; 6 Chemistry Department, College of Sciences, Taibah University, Madina Monawara 30002, Saudi Arabia; ohaddad@taibahu.edu.sa; 7 Chemistry Department, College of Sciences, Taibah University, Yanbu 30799, Saudi Arabia

**Keywords:** imine derivatives, thermal parameters, mesomorphic properties, conformational analysis, DFT

## Abstract

New three-ring ester/azomethine homologues series, (E)-4-((4-hydroxybenzylidene)amino)phenyl 4-(alkoxy)benzoate **I***n*, were prepared and their properties were investigated experimentally and theoretically. FT-IR, NMR, and elemental analyses were used to confirm the chemical structures of the synthesized compounds. The mesomorphic activities of the planned homologues were evaluated using differential scanning calorimetry (DSC) and polarized optical microscopy. All of the homologous examined were found to have non-mesomorphic properties. Theoretical calculations using the density functional theory (DFT) were used to validate the experimental data and determine the most stable conformation of the synthesized compounds. All calculated conformers’ thermal properties, dipole moments, and polarizability were discussed. The results show that the terminal alkoxy chain length affects the thermal parameters of the conformers. The correlations between these parameters’ values and the conformer type were demonstrated. The base component was expected to be in two conformers according to the orientation of the N atom of imine-linkage. DFT calculations revealed the more probable of the two possible conformers, and the incorporation of the alkoxy terminal chain in one position affect its geometrical and mesomerphic characteristics.

## 1. Introduction

Schiff bases are important chemical compounds that can be made by reacting an aldehyde or ketone with a primary amine, resulting in the synthesis of azome-thine (also known as imine) due to the release of a water molecule [1]. Imines and hydrazones are a class of organic compounds that have a wide range of applications in a variety of fields, including biological, analytical, and inorganic chemistry. Due to a wide range of biological activities, such as analgesic, antioxidant, antimicrobial, anticancer, anticonvulsant, antitubercular, anthelmintic, and anti-inflammatory activities, Schiff bases have gained popularity in the medicinal and pharmaceutical fields [2,3,4,5,6,7,8,9,10,11,12,13,14,15]. Schiff bases are also utilized as catalysts, organic synthesis intermediates, pigments, dyes, corrosion inhibitors and polymer stabilizers, [16,17,18]. Moreover, they influenced the development of coordination chemistry and were pivotal in the development of inorganic biochemistry and optical materials [19]. The use of imine derivatives in numerous processes encouraged researchers to produce novel heterocyclic/aryl Schiff bases for the creation of new environmentally friendly technology [20].

Because of their unique mesomorphic capabilities, azomethine/ester homologous series of liquid crystals (LCs) have been studied extensively to determine the relationship between chemical structure and mesomorphic properties [21,22,23,24,25]. In general, an organic compound’s mesophase stability and temperature ranges are determined significantly by its molecular shape, with even little changes in molecular geometry resulting in significant changes in mesomorphic behavior [26]. Within the hard core of the molecule, the Schiff base (azomethine group) is employed as a connecting group. Despite having a stepped core structure, the azomethine group retains molecular linearity, which makes the molecule more stable and, in most cases, allows the formation of the mesophase [27].

The phenolic derivatives have extensive importance in many different studies [28]. It was found that a stronger hydrogen-bonding between phenol and pyridine components in many complexes can be formed and promotes liquid-crystal behavior. In the case of acids moieties, a linear dimer is formed [29,30]. However, for phenols the hydrogen-bond complex is not linear and so does not promote liquid crystallinity [31]. The basic symmetrical and nonsymmetrical configurations of the designed molecule are affected by the number of aromatic units, the length of terminal chains, the alteration of polar spacers in elongating wings, and the mesomeric character of the central molecular core [32]. There have also been reports on the materials with two and three rings, as well as non-symmetrical molecular shapes and a core unit with one or two different linkages [33,34]. We recently looked into the thermal stability of symmetrical materials having the azomethine linkage as the central connecting group, 4-alkoxybenzoyloxy 4’-phenylmethineazophenyloxy 4”-alkoxybenzoate, in the mesophase [35]. Recently, it has been shown that the possible orientation of atoms in molecules is employed to modify existing functions, thereby introducing a new geometrical characteristic to the organic system.

**Figure 1:**
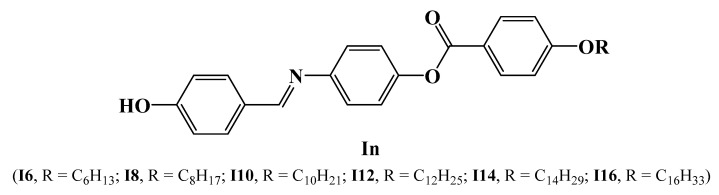
Molecular structure of investigated series **I***n*.

The goal of our work is to study the mesomorphic properties, and geometrical parameters of synthesized three-ring imine derivatives, namely (E)-4-((4-hydroxybenzylidene)amino)phenyl 4-(alkoxy)benzoate **I***n* (Figure 1). The work also includes theoretical analyses using DFT and experimental observations to explain how the varied orientations of the imine linkage included within the expected conformers affect these variables. Finally, we intend to study the effect of the terminal flexible chain on the mesomorphic behavior as well as geometrical and thermal parameters.

## 2. Experimental

### Synthesis

**Scheme 1:**
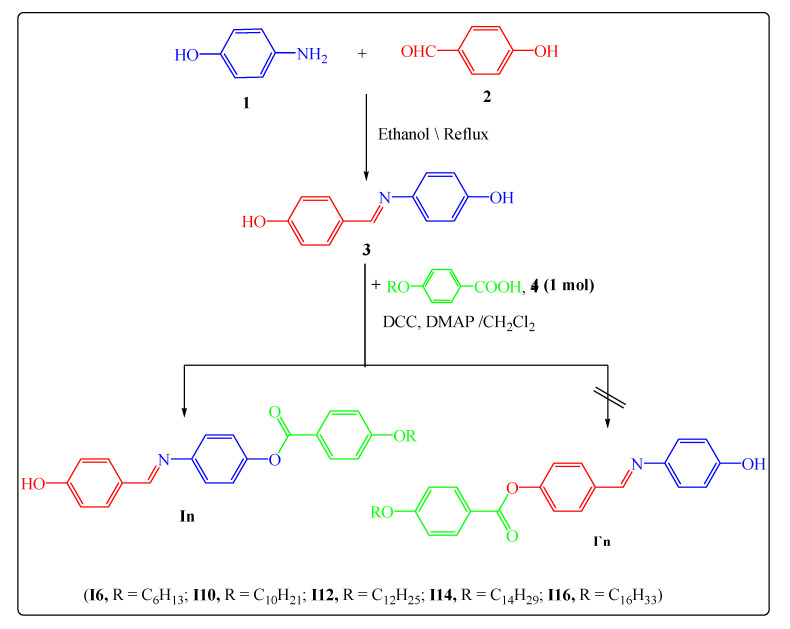
Synthetic method of series **I***n*.

The strategy utilized to construct the title compounds **I***n* includes two sequentially steps that start with the synthesis of (E)-4-((4-hydroxybenzylidene)amino)phenol **3** [34], by reacting 4-hydroxy aniline **1** with the respective 4-hydroxybenzaldehyde **2**. Compound **3** could then be converted to their corresponding targets (E)-4-((4-hydroxybenzylidene)amino)phenyl 4-(alkoxy)benzoate **I***n* through reaction with 4-alkoxybenzoic **4** in dry methylene chloride containing *N*,*N′−*dicyclohexylcarbodiimide (DCC) and catalytic amounts of 4*−*dimethylaminopyridine (DMAP) (Scheme 1).

The analyses results of products **I***n* are listed below:

**(E)-4-((4-hydroxybenzylidene)amino)phenyl 4-(hexyloxy)benzoate I***6*:

Yield: 89.0%; m.p. 124 °C, ^1^H-NMR (600 MHz, CDCl_3_): *δ*/ppm: 0.82 (t, 3H, CH_3_), 1.21–1.42 (m, 6H, CH_3_(CH_2_)_3_CH_2_CH_2_O-), 1.74–1.76 (m, 2H, CH_3_(CH_2_)_3_CH_2_CH_2_O-), 3.97 (t, 2H, CH_3_(CH_2_)_3_CH_2_CH_2_O-), 6.90–6.91 (d, 2H, Ar−H), 7.16–7.21 (d, 4H, Ar−H), 7.72 (d, 2H, Ar−H), 8.08–8.10 (d, 4H, Ar−H), 8.38 (s, 1H, CH=N), 9.90 (s, 1H, OH); FTIR (ύ, cm^−1^): 3417 (OH), 2928, 2832 (CH2 Stretching), 1729 (C=O), 1605 (C=N), 1573 (C=C), 1459 (C-O_Asym_), 1252 (C-O_Sym_). Anal. Calcd. for C_26_H_27_NO_4_ (417.50): C, 74.80; H, 6.52; N, 3.35. Found: C, 74.71; H, 6.46; N, 3.25%.

**(E)-4-((4-hydroxybenzylidene)amino)phenyl 4-(decyloxy)benzoate I***10*:

Yield: 88.0%; m.p. 87 °C, ^1^H-NMR (600 MHz, CDCl_3_): *δ*/ppm: 0.80–0.82 (t, 3H, CH_3_), 1.20–1.43 (m, 14H, CH_3_(CH_2_)_7_CH_2_CH_2_O-), 1.72–1.77 (m, 2H, CH_3_(CH_2_)_7_CH_2_CH_2_O-), 3.84–4.08 (t, 2H, CH_3_(CH_2_)_7_CH_2_CH_2_O-), 6.89–6.91 (d, 2H, Ar−H), 7.16–7.33 (d, 4H, Ar−H), 7.64 (d, 2H, Ar−H), 8.03–8.08 (d, 4H, Ar−H), 8.40 (s, H, CH=N), 9.92 (s, 1H, OH). ^13^C-NMR (600 MHz, CDCl_3_): *δ*/ppm: 14.14 (CH_3_), 22.71, 26.00, 29.12, 29.38, 29.58, 29.68, 29.70, 31.95, 68.35 (CH_2_), 110.64, 114.32, 121.16, 121.51, 121.82, 122.47, 123.24, 132.17, 132.30, 132.49, 132.54, 134.96, 143.03, 149.29, 149.40, 152.03, 159.61, 163.62, 164.25 (Ar-C and C=N), 165.05 (C=O); FTIR (ύ, cm^−1^): 3423 (OH), 2937, 2835 (CH2 Stretching), 1731 (C=O), 1613 (C=N), 1566 (C=C), 1461 (C-O_Asym_), 12547(C-O_Sym_). Anal. Calcd. for C_30_H_35_NO_4_ (473.60): C, 76.08; H, 7.45; N, 2.96. Found: C, 76.13; H, 7.36; N, 2.80%.

**(E)-4-((4-hydroxybenzylidene)amino)phenyl 4-(dodecyloxy)benzoate I***12*:

Yield: 89.8%; m.p. 127 °C, ^1^H-NMR (600 MHz, CDCl_3_): *δ*/ppm: 0.79–0.82 (t, 3H, CH_3_), 1.18–1.570 (m, 18H, CH_3_(CH_2_)_9_CH_2_CH_2_O-), 1.73–1.77 (m, 2H, CH_3_(CH_2_)_9_CH_2_CH_2_O-), 4.06–4.08 (t, 2H, CH_3_(CH_2_)_9_CH_2_CH_2_O-), 6.72–6.91 (d, 2H, Ar−H), 7.33 (d, 4H, Ar−H), 7.46 (d, 2H, Ar−H), 8.04–8.07 (d, 4H, Ar−H), 8.39 (s, 1H, CH=N). 9.90 (s, 1H, OH); FTIR (ύ, cm^−1^): 3421 (OH), 2933, 2852 (CH2 Stretching), 1726 (C=O), 1609 (C=N), 1562 (C=C), 1463 (C-O_Asym_), 1244 (C-O_Sym_). Anal. Calcd. for C_32_H_39_NO_4_ (501.66): C, 76.61; H, 7.84; N, 2.79. Found: C, 76.59; H, 7.75; N, 2.60%.

**(E)-4-((4-hydroxybenzylidene)amino)phenyl 4-(tetradecyloxy)benzoate I***14*:

Yield: 91.8%; m.p. 93 °C, Yield: 89.8%; m.p. 98.7 °C, ^1^H-NMR (600 MHz, CDCl_3_): *δ*/ppm: 0.79–0.81 (t, 3H, CH_3_), 1.19–1.41 (m, 22H, CH_3_(CH_2_)_11_CH_2_CH_2_O-), 1.72–1.85 (m, 2H, CH_3_(CH_2_)_11_CH_2_CH_2_O-), 3.84–3.98 (t, 2H, CH_3_(CH_2_)_11_CH_2_CH_2_O-), 6.89–6.90 (d, 2H, Ar−H), 7.15–7.33 (d, 4H, Ar−H), 7.64 (d, 2H, Ar−H), 8.07–8.09 (d, 4H, Ar−H), 8.37 (s, 1H, CH=N), 9.93 (s, 1H, OH).^13^C-NMR (600 MHz, CDCl_3_): *δ*/ppm: 14.13 (CH_3_), 22.70, 25.46, 25.64, 25.99, 29.37, 29.57, 29.60, 29.67, 29.69, 29.70, 31.94, 33.96, 68.35 (CH_2_), 110.63, 114.32, 121.14, 121.49, 121.82, 122.47, 123.22, 123.28, 132.29, 132.49, 134.97, 143.01, 149.27, 149.41, 152.02, 156.79, 159.61, 163.62, 164.27 (Ar-C and C=N), 165.06 (C=O); FTIR (ύ, cm^−1^): 3417 (OH), 2920, 2828 (CH2 Stretching), 1726 (C=O), 1602 (C=N), 1575 (C=C), 1466 (C-O_Asym_), 1246 (C-O_Sym_). Anal. Calcd. for C_34_H_43_NO_4_ (529.71): C, 77.09; H, 8.18; N, 2.64. Found: C, 77.00; H, 8.04; N, 2.52%.

**(E)-4-((4-hydroxybenzylidene)amino)phenyl 4-(hexadecyloxy)benzoate I***16*:

Yield: 90.8%; m.p. 99 °C, ^1^H-NMR (600 MHz, CDCl_3_): *δ*/ppm: 0.77–0.81 (t, 3H, CH_3_), 1.18–1.41 (m, 26H, CH_3_(CH_2_)_13_CH_2_CH_2_O-), 1.73–1.77 (m, 2H, CH_3_(CH_2_)_13_CH_2_CH_2_O-), 3.82–3.99 (t, 2H, CH_3_(CH_2_)_13_CH_2_CH_2_O-), 6.89–6.92 (d, 2H, Ar−H), 7.15–7.39 (d, 4H, Ar−H), 7.46 (d, 2H, Ar−H), 8.02–8.08 (d, 4H, Ar−H), 8.39 (s, 1H, CH=N). 9.95 (s, 1H, OH); FTIR (ύ, cm^−1^): 3412 (OH), 2919, 2839 (CH2 Stretching), 1728 (C=O), 1600 (C=N), 1566 (C=C), 1462 (C-O_Asym_), 1246 (C-O_Sym_). Anal. Calcd. for C_36_H_47_NO_4_ (557.76): C, 77.52; H, 8.49; N, 2.51. Found: C, 77.39; H, 8.35; N, 2.40%.

## 3. Results and Discussion

### 3.1. Mesomorphic Properties Investigations of Series ***I***n

**Figure 2:**
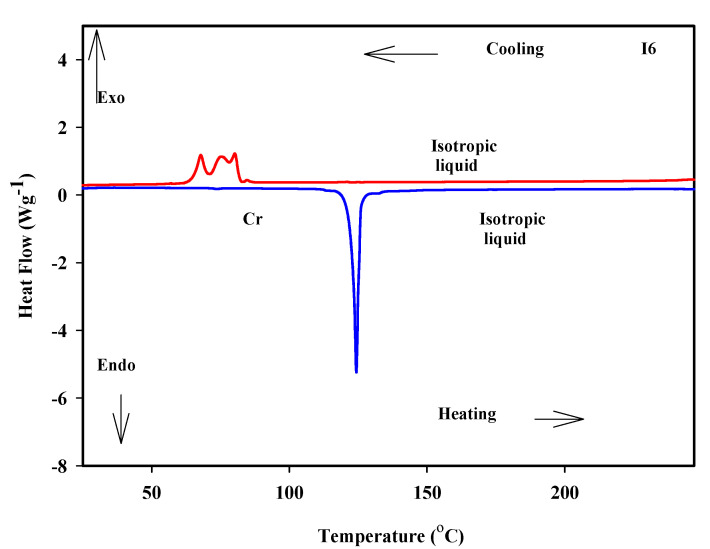
DSC thermogram of sample **I***6* on heating/ cooling cycles with rate 10 °C min^−1^.

**Figure 3:**
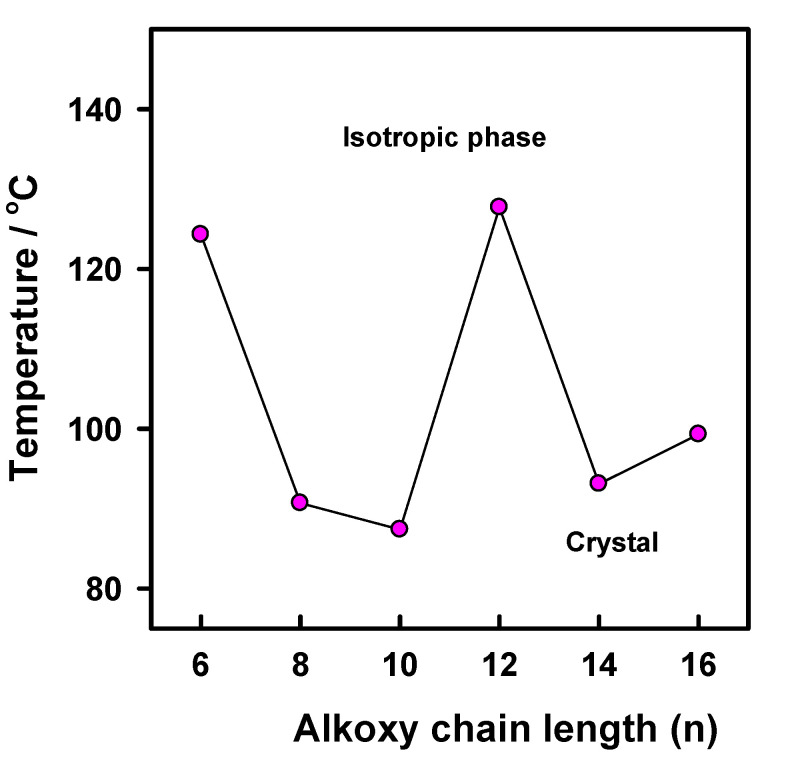
Dependency of alkoxy chain length (n) on the thermal properties of series **I***n*.

The current synthetic series (**I***n*) was investigated for its mesomorphic properties. Table 1 summarizes the results of the transition temperatures and enthalpies as obtained by DSC measurements. To evaluate the stability of the synthesized compounds, DSC results from the second heating/cooling cycles were estimated. The second heating scan was utilized to record all compounds' thermal characteristics. The DSC curve of the synthesized ester/azomethine homologue I*6* through heating and cooling scans is shown in Figure 2. On heating, the homologous revealed only one endothermic peak corresponding to the crystal-to-isotropic transition, whereas cooling revealed one reversed exothermic peak also, as shown in Figure 2. The POM textures also confirmed the DSC data. All examined compounds of the present series (**I***n*) are nonmesomorphic with high thermal stabilities, as shown in Table 1 and Figure 3. Moreover, the melting points of all prepared derivatives highlighted in Table 1 and Figure 3 follow a random pattern. In general, the polarity and/or polarizability of the mesogenic core of the molecule play the most critical roles in determining mesophase behavior. The length of the terminal group, on the other hand, has a considerable influence on the kind and stability of the produced mesophases. Finally, geometrical characteristics such as dipole moment, polarizability, and molecular architecture are important in the formation of any mesomorphic phases.

Many properties of linear molecules, such as polarizability, dipole moment, aspect ratio, and competitive contact between terminal moieties, influence their mesomorphic properties, as established in the literature. The mesomeric configurations are known to alter molecular geometry and this has a significant impact on the molecular–molecular interactions. As shown in our experiments, molecular packing between present investigated molecules affects the thermal stabilities and this in return led to the non-mesomorphic properties. This assertion quite agrees with the reports on some phenolic derivatives [35].

### 3.2. Theoretical Calculations

#### 3.2.1. Computational Details

Each set of the isomers investigated were completely optimized to global minimum without geometrical restraints by GAUSSIAN 09 program [36]. The state of their convergence was examined via wavenumber calculation which predicted real value for all the frequencies. Furthermore, the frontier molecular orbitals and the molecular electrostatic potential surfaces were obtained from the formatted check (Fchk) file of the optimized structures. All the density functional theory (DFT) calculations were conducted using the B3LYP method [37,38] with the basis set of 6–31 G (d,p) which has been found to give results that are in fair agreement with the experimental data for relatively large molecules at a comparably cheap cost [39,40,41,42,43,44].

#### 3.2.2. Relative Stability

**Figure 4:**
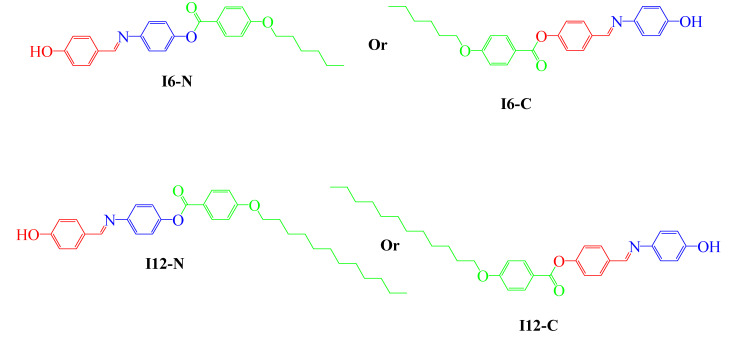
Configurationally isomers of two possible conformers of **I***6* and **I***12* derivatives as examples.

Based on the orientation of imine linkage, two possible conformers of isomers can exist as shown in Figure 4 obtained from the DFT calculation. Moreover, the presence and the nature of substituents on phenyl could play appreciable role in the stability of compounds. This assertion could be inferred from the result presented in Table 2, for which the isomers **I***n* series were generally predicted to be more stable than **I’***n* counterparts by approximately 1 kcal/mol. The –OH substituent and imine linkage are both activating groups that inductively donate electron to the phenyl system. Their presence together in the first phenyl part of the compounds would increase the electron pool in the phenyl system and this could have resulted in a relatively high repulsion, that eventually led to the lower stability recorded for the **I’***n* isomers. On the part of **I***n* isomers, the presence of the ester substituent, an electron withdrawing group on the second phenyl directly linked with the imine linkage aided their stability as it decreases the electron density on the phenyl group. Moreover, the similar relative energy difference calculated for the corresponding members in **I***n* and **I’***n* series suggests that their relative stability is less sensitive to the size of terminal alkoxy group.

#### 3.2.3. Reactivity Parameters

**Figure 5:**
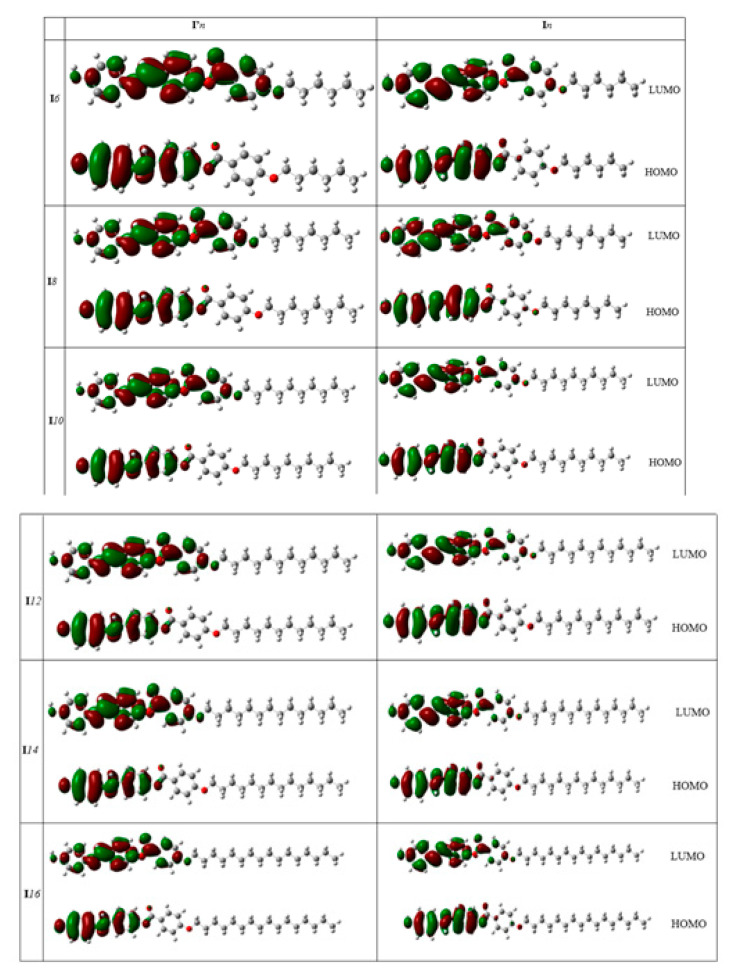
FOM’s calculated at B3LYP/6-31G(d.p) for investigated series **I***n*.

**Figure 6:**
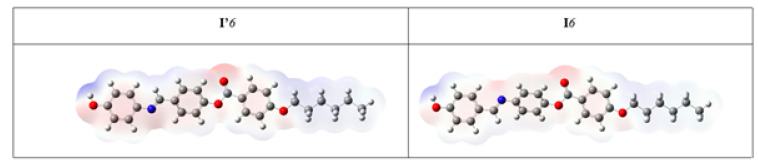
MEP calculated at B3LYP/6-31G(d.p) for the isomer **I***6*.

The reactivity of compounds is usually inferred from the HOMO-LUMO energy gap. The lower this gap the more reactive a molecule would be [40]. In addition, ionization potential (I.P) and electron affinity (EA) are other parameters that can attest to the reactivity of compounds [40,41]. These parameters were computed for the understudied isomers to determine their reactivity and the results are highlighted in Table 3. The **I’***n* isomers were predicted to be generally more reactive than their corresponding **I***n* isomers owing to the lower HOMO and LUMO energy gap. The higher energy gap calculated for the **I***n* isomers is consistent with the relative stability as this attests to their higher stability over the **I’***n* derivatives [41]. On the part of other reactivity indicators, the slightly lower value of ionization potential but higher value for the electron affinity computed for the **I’***n* isomers over the corresponding **I***n* isomers, suggests that the **I’***n* configurations are both slightly more basic and acidic that the **I***n* derivatives. The **I’***n* isomers being more basic and acidic than the **I***n* conformations is consistent with the reports in literature [41,42]. Analysis of these reactivity indicators shows that the compound’s reactivity is not significantly influenced by the length of terminal alkoxide as close values were predicted for the member of each set of the isomers. In the case compound polarity, the greater dipole moment together with the isotropic polarizability recorded for the **I’***n* isomers suggests higher polarity over the **I***n* counterparts. [43,44]. On the other hand, the frontier molecular orbitals study portrayed in Figure 5 showed similar molecular distributions between the two isomers at the HOMO level as well as that of the LUMO (FMO’s). This could be attributed to the little difference between the corresponding HOMO and LUMO energy levels for each member of the two the isomers [40]. On the part of the HOMO, the electron clouds were evenly distributed over the oxygen atom of –OH group, carbon atoms and the π- system of the first two phenyl rings as well as the –N=CH– linkage between them. However, the third phenyl ring and its attached terminal alkyl group were highly electron deficient. The electron deficiency of alkyl groups could be attributed to the resultant effect of the O-C=O linkage, which is an electron withdrawal that resonantly stabilizes the phenoxide ion to which it is attached. This makes the phenoxide more acidic and, as such, causes electrons withdrawal from alkyl groups. In the case of LUMO, the oxygen atom of –OH group has lower electron density compared to that of HOMO while the electron clouds are only distributed over the carbon atoms of the first phenyl ring. Furthermore, the electron clouds distribution covers carbon atoms and the π- system of the second phenyl ring, the carbonyl part of the –OCO– between the second and third phenyl rings together with the carbon atoms of the third phenyl ring. On the part of the molecular electrostatic potential (MEP) presented in Figure 6, the red cloud above the carbonyl and phenolic oxygen atoms indicates low electrostatic potential but high electron density. On the other hand, the blue cloud over the phenolic hydrogen portrays high electrostatic potential with low electron density [45,46,47,48,49]. 

#### 3.2.4. Calculated Energies

The magnitude of any of thermodynamic dynamic parameters as well as energy of a system is size-dependent. This assertion is reflected in the results presented in Table 4 for which the values of all the calculated parameters increase with the increasing chain length of terminal alkoxy chain [44,45,46,47,49]. Moreover, the similar values predicted for the energy indicators of the corresponding members of the two isomers is due fact that they have the same molecular formula but only differ in configurations which do not significantly affect the energy. 

## 4. Conclusions

New imine homologues series, (E)-4-((4-hydroxybenzylidene)amino)phenyl 4-(alkoxy)benzoate, were synthesized and examined via experimental and theoretical approaches. Their thermal and mesomorphic behaviors were investigated using DSC and POM. All new compounds were found to be non-mesomorphic. DFT theoretical calculations were based on two conformers of each derivative. The computational study revealed that the orientation of imine linkage is vital to the isomer stability while their thermal properties were predicted to be size-dependent.

## Figures and Tables

**Table 1:** DSC transition temperatures (°C) and enthalpy of transition **ΔH**, kJ/mole for series **I***n*.

Compound	*T*_Cr-I_	Δ*H*_Cr-I_
I*6*	124.3	43.73
I*8*	90.7	40.29
I*10*	87.4	42.80
I*12*	127.7	56.2
I*14*	93.1	45.20
I*16*	99.3	54.90

Cr-I = solid to the isotropic liquid phase transition.

**Table 2:** Relative energies for series **I***n* and their isomers in kcal/mol.

Isomer	I*6*	I*8*	I*10*	I*12*	I*14*	I*16*
**I** *n*	0	0	0	0	0	0
**I’** *n*	0.74	0.71	0.74	0.74	0.74	0.71

**Table 3:** Reactivity parameters calculated at B3LYP/6-31G(d,p).

Isomer	E_HOMO_ (eV)		E_LUMO_ (eV)		ΔE (eV)		Dipole Moment (Debye)		I.P (eV)		E.A (eV)		Isotropic Polarizability (Bohr3)	
	**I** *n*	**I’** *n*	**I** *n*	**I’** *n*	**I** *n*	**I’** *n*	**I** *n*	**I’** *n*	**I** *n*	**I’** *n*	**I** *n*	**I’** *n*	**I** *n*	**I’** *n*
**I** *6*	−5.533	−5.409	−1.397	−1.506	4.136	3.903	1.842	4.0524	5.533	5.409	1.397	1.506	347.76	350.27
**I** *8*	−5.531	−5.409	−1.396	−1.504	4.135	3.904	1.8823	4.0943	5.531	5.409	1.396	1.504	371.19	373.63
**I** *10*	−5.544	−5.409	−1.395	−1.504	4.149	3.905	1.854	4.1151	5.544	5.409	1.395	1.504	393.15	396.75
**I** *12*	−5.544	−5.409	−1.394	−1.503	4.150	3.905	1.8637	4.1272	5.544	5.409	1.394	1.503	416.11	419.74
**I** *14*	−5.545	−5.409	−1.394	−1.503	4.151	3.906	1.8684	4.1342	5.545	5.409	1.394	1.503	438.98	442.66
**I** *16*	−5.530	−5.401	−1.396	−1.519	4.135	3.883	1.9253	4.1305	5.530	5.401	1.396	1.519	463.15	466.97

**Table 4:** Zero-point energy and thermodynamic parameters for series **I***n*, calculated at B3LYP/6-31G(d,p).

Isomer	ZPE (kcal/mol)		Thermal (kcal/mol)		Enthalpy (kcal/mol)		Gibbs (kcal/mol)		Entropy (cal/mol.K)	
	**I** *n*	**I’** *n*	**I** *n*	**I’** *n*	**I** *n*	**I’** *n*	**I** *n*	**I’** *n*	**I** *n*	**I’** *n*
**I** *6*	299.466	299.473	317.878	317.865	318.470	318.458	257.610	258.042	204.125	202.637
**I** *8*	335.279	335.256	355.383	355.355	355.976	355.947	291.020	291.237	217.865	217.036
**I** *10*	371.030	371.035	392.831	392.841	393.423	393.433	324.303	324.401	231.829	231.534
**I** *12*	406.805	406.815	430.315	430.327	430.907	430.919	357.429	357.555	246.449	246.064
**I** *14*	442.582	442.595	467.801	467.813	468.393	468.405	390.546	390.715	261.100	260.576
**I** *16*	478.457	478.409	505.372	505.343	505.964	505.936	423.924	423.811	275.164	275.446

## Data Availability

The data presented in this study are available on request from corresponding authors.
